# Fat Mass Is Associated with Serum 25-Hydroxyvitamin D Concentration Regardless of Body Size in Men

**DOI:** 10.3390/nu10070850

**Published:** 2018-06-29

**Authors:** Kyung-Jin Yeum, Bess Dawson-Hughes, Nam-Seok Joo

**Affiliations:** 1 Department of Integrated Biosciences, College of Biomedical and Health Science, Konkuk University, Chungju-si 27478, Korea; kyeum@kku.ac.kr; 2 Jean Mayer US Department of Agriculture Human Nutrition Research Center on Aging, Tufts University, Boston, MA 02111, USA; bess.dawson-hughes@tufts.edu; 3 Ajou University School of Medicine, Suwon 16499, Korea

**Keywords:** body mass index, 25-hydroxyvitamin D, fat mass, body weight

## Abstract

There are no large community-based studies examining the association of body size vs. body fat with vitamin D status. Association of serum 25-hydroxyvitamin D (25OHD) with body weight and subcategories of body weight defined by fat mass were evaluated in a large, free living population. Out of a total of 29,235 subjects from the 2008–2010 Korean National Health and Nutrition Examination Survey, the relevant data included 6458 subjects over 50 years of age who were analyzed cross-sectionally. Serum 25OHD concentrations were compared in men (*n* = 3164) and in women (*n* = 3294) by tertiles of body weight and body fat mass, as measured by Dual-energy X-ray Absorptiometry (DXA) within sex-specific tertiles of body weight. Serum 25OHD was weakly inversely correlated with body weight in the men and the women after adjustment for age (*r* = −0.075 and −0.073, respectively, *p* < 0.001 for both). Within each tertile of body weight, serum 25OHD decreased progressively as fat mass increased in men. This pattern was similar in the women but not consistently significant. Whereas body weight predicted a small decrease in serum 25OHD in the men and the women, greater adiposity, for any given weight, predicted larger decreases in the men, but not consistently in women.

## 1. Introduction

Vitamin D deficiency is associated with various chronic diseases such as Type 2 diabetes [1], cardiovascular disease [2], some cancers [3], and poor bone health [4]. Understanding the factors affecting vitamin D status is critical towards the development of interventions for such diseases. When defining vitamin D deficiency, serum 25-hydroxyvitamin D (25OHD), a circulating form of vitamin D, is considered the gold standard as a marker for vitamin D status [5]. Serum 25OHD concentration, however, can be influenced by season [6], skin type or ethnicity [7], total outdoor activity [8], and dietary intake or supplementation [9].

Because vitamin D is a fat-soluble vitamin, its status can be affected by the amount of total body fat (known as sequestration). The amount of vitamin D stored in adipose tissue has been shown to be substantially increased after long-term supplementation with high doses of vitamin D [10]. Accordingly, serum 25OHD status was reported to be inversely associated with body fat in older adults [11], as well as percent body fat in overweight and obese individuals [12]. In addition to older adults, even in young women [13] and children [14], vitamin D insufficiency was associated with increased body fat or higher adiposity. In particular, serum 25OHD status was reported to be strongly associated with both subcutaneous and visceral adipose tissues measured by multidetector computed tomography [15]. On the other hand, low vitamin D status in obese individuals may also be explained by dilution of ingested or cutaneously synthesized vitamin D, thus implicating body weight as a major factor affecting serum 25OHD status [16]. Therefore, the aim of the current study was to determine the association of serum 25OHD concentration with body weight and subcategories of body weight as defined by fat mass in a large, free living population.

## 2. Subjects and Methods

### 2.1. Data Sources

**Figure 1:**
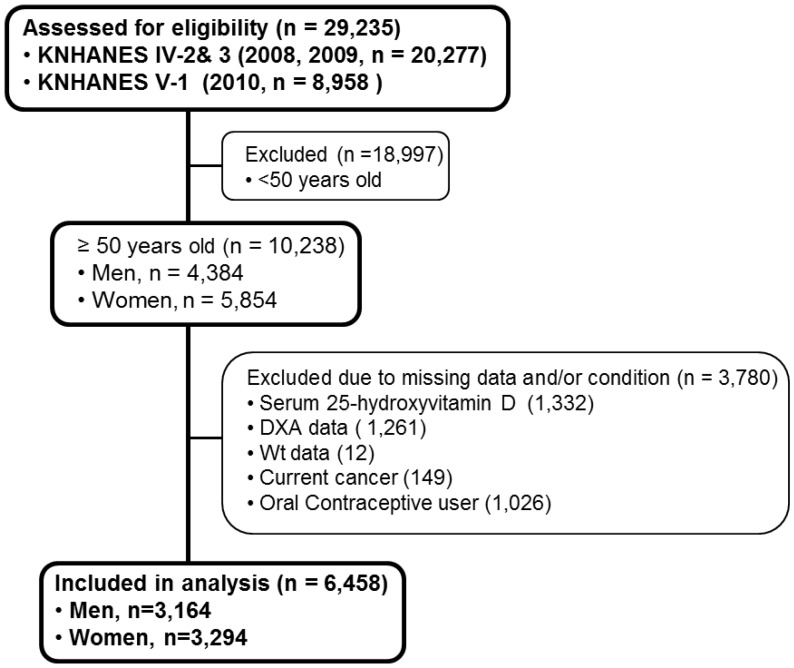
Flow diagram of subject inclusion and exclusion in the Korean National Health and Nutrition Examination Surveys (KNHANES IV-2, 3 & V-1). DXA, Dual-energy-X ray-Absorptiometry; Wt, body weight.

The Korean National Health and Nutrition Examination Survey (KNHANES), conducted periodically by the Korea Centers for Disease Control and Prevention since 1998, provides comprehensive information on the health status, health behavior, nutritional status and socio-demographics of 600 national districts in Korea. The Koreans are an ethnically homogenous group sharing similar skin color. The health interview survey in KNHANES was conducted through face-to-face interviews at the homes of participants by trained interviewers. Data from the fourth (IV-2, 3, 2008, 2009) and fifth (V-1, 2010) KNHANES data samplings containing serum 25OHD concentration, body weight, and total body fat data were utilized in this cross-sectional analysis. From an initial total of 29,235 male and female, 10,238 subjects aged over 50 years were evaluated. We focused on subjects aged 50 years and older since the incidence of overweight and obesity is relatively higher in people older rather than younger in this population. Of the evaluated subjects, 3780 subjects were excluded for missing data including body serum 25OHD (1332 subjects), DXA (1261 subjects), body weight (12 subjects), as well as current cancers (149 subjects) and oral contraceptive users (1026 subjects). A final 6458 subjects (3164 men and 3294 women) were used in this analysis (Figure 1). All participants provided written informed consent before the survey.

### 2.2. Biochemistry and DXA Measurements

Blood samples, after an 8 h fast, were collected year-round, similar number of subjects in each season. Upon collection, these samples were immediately processed, refrigerated, and transported in cold storage to the central testing institute (NeoDin Medical Institute, Seoul, South Korea) and analyzed within 24 h. Serum 25(OH)D concentration was measured with a radioimmunoassay (RIA) kit (DiaSorin Inc., Stillwater, MN, USA) using a γ-counter (1470Wizard; PerkinElmer, Turku, Finland). The inter-assay coefficients of variation (CV) were 2.8–6.2% for the 2008–2009 samples and 1.9–6.1% for 2010 samples. Serum 25(OH)D was measured at the same institute which performed quality control every other week throughout the analysis period to minimize analytical variation. Body fat mass was measured by Dual-Energy X-ray Absorptiometry (DXA, DISCOVERY-W fan-beam densitometer, Hologic Inc., Marlborough, MA, USA) with CVs of 1.9% and 2.5%, respectively. Total calorie intake was assessed with a 24 h dietary recall questionnaire administered by a trained dietician. The results were calculated using the Food Composition Table developed by the National Rural Resources Development Institute (7th revision) [17]. Contents of dietary supplements were not documented in KNHANES.

### 2.3. Statistical Analyses

The complex sample analysis used for the KNHANES data for weighting all values followed guidance for statistics from the Korea Centers for Disease Control and Prevention. General characteristics of all study subjects including age, body weight, height, body mass index (BMI), total body fat mass, trunk fat mass, limb fat mass, lean body mass, serum 25OHD concentration, and total calorie intake were represented in men and women after data weighting. Partial correlation analyses were run to evaluate the associations of serum 25OHD concentration with body weight, height, BMI, waist circumference, total body fat mass, trunk fat mass, limb fat mass, and lean body mass in both genders after adjusting for age. In addition, multiple regression analysis with the dependent variable as 25OHD was conducted to determine the statistical significance of each independent variable such as total body fat mass, age, and sex. To evaluate the difference of each variable according to body weight, we divided the body weight of all subjects into tertiles and compared all variables by their tertiles using ANCOVA test in complex sample analysis. To determine the association of serum 25OHD concentration with total body fat mass, we also divided total body fat mass into tertiles and compared serum 25OHD concentration (dependent variable) by the tertiles of body weight (fixed factor) and total body fat mass (categorical factor) after age and height adjustments. In addition to the evaluation of total body fat mass with serum 25OHD concentration, trunk fat mass and limb fat mass with serum 25OHD concentration was also analyzed using the same method to find similar correlations. *P* for trend was used to assess the significance of all analysis. Data were analyzed using SPSS 19.0 (SPSS Inc., Chicago, IL, USA) to account for the complex sampling design.

## 3. Results

In this cross-sectional analysis, the data of 6458 subjects were included (3164 men and 3294 women over the age of 50 years). The mean age of men and women was 60.8 years and 62.3 years and the mean body mass index (BMI) was 23.9 kg/m^2^ and 24.2 kg/m^2^, respectively. Mean total body fat mass in man and women are in normal ranges as presented in Table 1. Mean serum 25OHD concentration was 21.6 ng/mL in men and 18.6 ng/mL in women, and daily calorie intake was 2166.7 kcal/day in men and 1566.4 kcal/day in women.

### 3.1. Weight, Fat and Lean Tissue Associations

**Figure 2:**
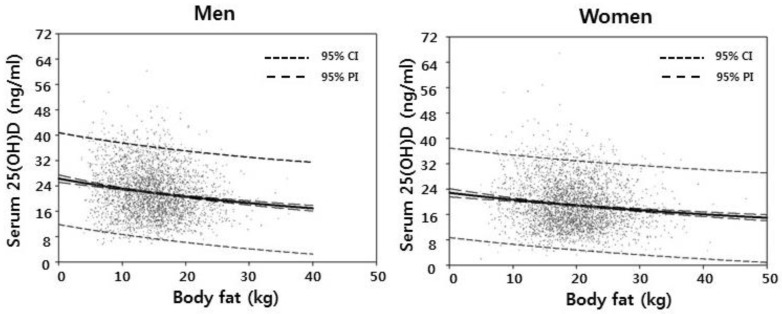
Regression lines of serum 25-hydroxyvitamin D concentration with total body fat in men and women. 95% CI means 95% Confidence Interval, 95% PI means 95% Prediction Interval, and 25(OH)D means 25-hyroxyvitamin D.

Considering that men and women are different in terms of body fat mass as shown in Figure 2, men and women were analyzed separately.

Serum 25OHD was significantly but weakly inversely correlated with body weight in men (*r* = −0.075, *p* < 0.001) and women (*r* = −0.073, *p* < 0.001), after adjustment for age (Table 2). Stronger inverse correlations were observed between serum 25OHD and total body fat in men and women (*r* = −0.154 and −0.116, respectively, *p* < 0.001 for both). Serum 25OHD was not significantly correlated with lean body mass in the men or the women (Table 2).

In addition, multiple regression analysis indicated that serum 25OHD concentration was significantly associated with total body fat mass (*p* < 0.001), age (*p* = 0.003), and sex (*p* < 0.001).

### 3.2. Fat Mass within Tertiles of Body Weight

**Figure 3:**
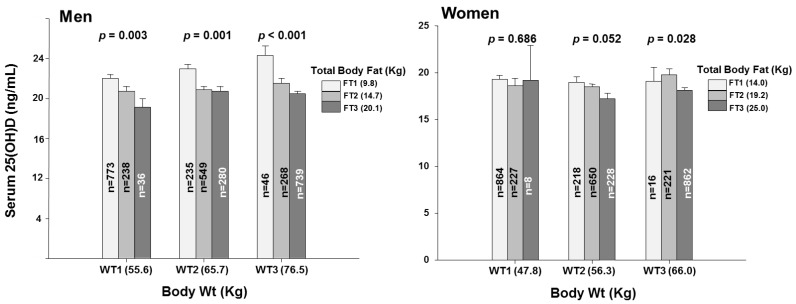
Serum 25-hydroxyvitamin D concentrations by total body fat tertiles within each body weight tertile categories. 25(OH), 25-hydroxyvitamin D; WT1, the first tertile of body weight (55.6 Kg, 31.9–61.6 Kg for men and 47.9 Kg, 32.3–52.7 Kg for women); WT2, the second tertile of body weight (65.7 Kg, 61.7–69.8 Kg for men and 56.3 Kg, 52.8–59.8 Kg for women); WT3, the third tertile of body weight (76.5 Kg, 69.9–108.6 Kg for men and 66.0 Kg, 59.9–100.0 Kg for women); Bar plots in which each total weight tertile categories represent tertiles of total body fat mass (FT1, the first tertile of total body fat mass, 9.8 Kg, 3.3–12.7 Kg for men and 14.0 Kg, 4.9–17.1 Kg for women; FT2, the second tertile of total body fat mass, 14.7 Kg, 12.7–16.5 Kg for men and 19.2 Kg, 17.1–21.4 Kg for women; FT3, the third tertile of total body fat mass, 20.1 Kg, 16.5–36.2 Kg for men and 25.0 Kg, 21.4–46.8 Kg for women). Data plotted represent mean and standard deviation. *p* for trend in same groups are from general lineal model in complex data analysis.

**Figure 4:**
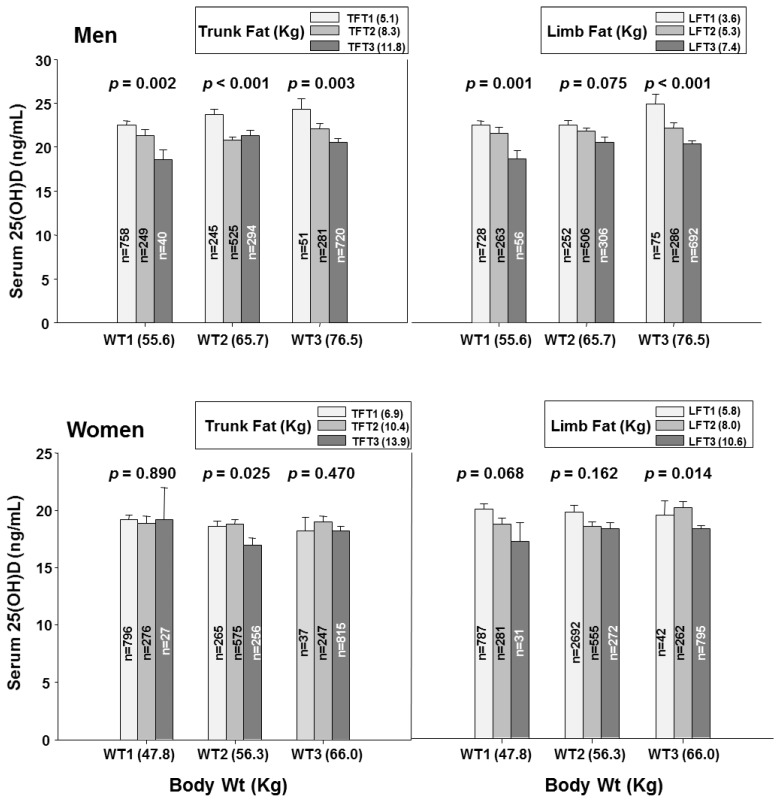
Serum 25-hydroxyvitamin D concentrations by trunk fat and limb fat tertiles within each body weight tertile categories. 25(OH), 25-hydroxyvitamin D; WT1, the first tertile of body weight (55.6 Kg, 31.9–61.6 Kg for men and 47.9 Kg, 32.3–52.7 Kg for women); WT2, the second tertile of body weight (65.7 Kg, 61.7–69.8 Kg for men and 56.3 Kg, 52.8–59.8 Kg for women); WT3, the third tertile of body weight (76.5 Kg, 69.9–108.6 Kg for men and 66.0 Kg, 59.9–100.0 Kg for women); Bar plots in which each body weight tertile categories represent tertiles of trunk fat mass (TFT1, the first tertile of trunk fat mass, 5.1 Kg, 1.3–6.9 Kg for men and 6.9 Kg, 1.9–8.9 Kg for women; TFT2, the second tertile of trunk fat mass, 8.3 Kg, 6.9–9.6 Kg for men and 10.4 Kg, 9.0–11.6 Kg for women; TFT3, the third tertile of trunk fat mass, 11.8 Kg, 9.6–20.9 Kg for men and 13.9 Kg, 11.6–25.5 Kg for women) and limb fat mass (LFT1, the first tertile of limb fat mass, 3.6 Kg, 1.1–4.6 Kg for men and 5.8 Kg, 1.9–7.1 Kg for women; LFT2, the second tertile of limb fat mass, 5.3 Kg, 4.6–6.1 Kg for men and 8.0 Kg, 7.1–8.9 Kg for women; LFT3, the third tertile of limb fat mass, 7.4 Kg, 6.1–14.4 Kg for men and 10.6 Kg, 8.9–20.2 Kg for women). Data plotted represent mean and standard deviation. *p* for trend in same groups are from general lineal model in complex data analysis.

To gain insight into whether the association of body fat with serum 25OHD concentration was dependent or independent of body weight, we examined the associations of serum 25OHD concentration with tertiles of body fat within low, middle, and high tertiles of body weight (Figure 3). In men, serum 25OHD concentration declined progressively and significantly with increasing fat mass within each of the weight tertiles. The same pattern was seen for truncal and for limb fat in the men (Figure 4). In women, the patterns ware generally similar but less pronounced and with only scattered statistical significance (Figure 3 and Figure 4).

Notably, serum 25OHD declined numerically but not significantly across the weight tertiles whereas, as expected, fat and lean tissue mass increased significantly (Table 3).

The same pattern was seen for truncal and for limb fat mass.

**Figure 5:**
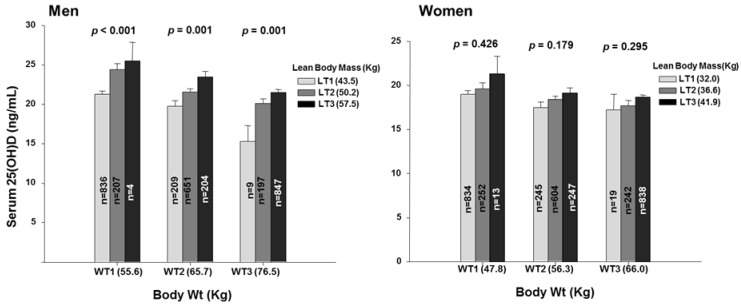
Serum 25-hydroxyvitamin D concentrations by lean body mass tertiles within each body weight tertile categories. 25(OH), 25-hydroxyvitamin D; WT1, the first tertile of body weight (55.6 Kg, 31.9–61.6 Kg for men and 47.9 Kg, 32.3–52.7 Kg for women); WT2, the second tertile of body weight (65.7 Kg, 61.7–69.8 Kg for men and 56.3 Kg, 52.8–59.8 Kg for women); WT3, the third tertile of body weight (76.5 Kg, 69.9–108.6 Kg for men and 66.0 Kg, 59.9–100.0 Kg for women); Bar plots in which each body weight tertile categories represent tertiles of lean body mass (LT1, the first tertile of lean body mass, 43.5 Kg, 26.7–47.5 Kg for men and 32.0 Kg, 24.2–34.8 Kg for women; LT2, the second tertile of lean body mass, 50.2 Kg, 47.6–53.0 Kg for men and 36.6 Kg, 34.8–38.4 Kg for women; LT3, the third tertile of lean body mass, 57.5 Kg, 53.1–76.4 Kg for men and 41.9 Kg, 38.4–56.2 Kg for women). Data plotted represent mean and standard deviation. *p* for trend in same groups are from general lineal model in complex data analysis.

In addition, the associations of serum 25OHD concentration with tertiles of lean body mass within low, middle, and high tertiles of body weight were examined as presented in Figure 5. Serum 25OHD increased progressively and significantly with increasing lean body mass within each of the weight tertiles in the men.

## 4. Discussion

In this large, cross-sectional study, serum 25OHD concentration was weakly inversely associated with weight in the men and the women, as has been widely reported [18,19]. Our finding that, in men, serum 25OHD was strongly inversely associated with total (and truncal, and limb) fat within the low as well as the middle and high weight groups is consistent with body fat as an independent determinant of serum 25OHD concentration. This finding argues against dilution as the sole cause of the low 25OHD levels seen in larger adults [16] and instead suggests that men and women with large amounts of body fat are at increased risk of vitamin D deficiency.

The reason for the observed attenuated associations of serum 25OHD with fat mass in women compared with men is uncertain, and it is unclear that there are in fact significant gender differences in the associations of 25OHD with fat mass. An independent association between serum 25OHD and total body and regional fat mass in non-osteoporotic, overweight, postmenopausal Greek women (*n* = 112) has been reported [20]. Furthermore mean serum 25OHD concentration was also negatively associated with total body fat percentage and total body fat in young Hispanic women [21]. A previous meta-analysis [22] reporting the relationship of vitamin D status with body mass index and with body fat [23] is also in line with the current study. However, these studies are limited in distinguishing the effects of body size and fat mass on vitamin D status.

There are several potential explanations for how fat tissue may influence circulating 25OHD levels. Vitamin D insufficiency has been associated with increased fat infiltration in muscle in healthy young women [24]. The link between obesity and low circulating 25OHD concentration [25] could be explained through adipose sequestration of vitamin D [10] and/or increased catabolism of vitamin D due to the local action of 24-hydroxylase that has been found in human adipose tissue [26]. Another possible explanation is that synthesis of 25OHD by the liver may be reduced in obese relative to lean individuals [27]. In addition, lifestyle can be a determinant of 25OHD, with less sunlight exposure leading to reduced 25OHD synthesis in the skin [28]. However, we have not found that reduced sun exposure accounts for lower serum 25OHD levels in obese adults [29]. As for fat distribution, our study showed that trunk fat mass and limb fat mass gave a similar contribution to low serum 25OHD concentration in contrast to other studies showing a distinct role of visceral adipose in determining plasma 25OHD concentration compared to other adipose tissue compartments [15,30,31,32].

Changes of serum 25OHD concentration after body weight changes may give a clue to how serum 25OHD is related to body fat. The Insulin Resistance Atherosclerosis Family Study examining the relationship between 25OHD and adiposity in Hispanic and African-Americans showed an inverse association between vitamin D concentrations and baseline BMI, in subcutaneous adipose tissue as well as visceral adipose tissue. However, this trend was no longer observed to exist after a 5-year change in adiposity [33]. On the other hand, weight loss of 10% by a low-calorie diet resulted in an increase of serum 25OHD concentration [34]. It may be possible that an acute increase of serum 25OHD concentrations comes from stored vitamin D in adipose tissue. In particular, in morbidly obese patients waiting for bariatric surgery, vitamin D deficiency is frequently seen where serum 25OHD concentration increases temporarily at 1 month and then decreases during the 24 months following Roux-en-y gastric bypass (RYGB) surgery [35]. There is still no clear consensus whether adipose tissue regulates serum 25OHD concentration according to body fat changes. Even though adipose tissue is considered as a large vitamin D reservoir and may be an important contributing factor to circulating 25OHD, metabolism 25OHD metabolism in adipocytes and its subsequent effects on serum 25OHD remain unknown.

## 5. Conclusions

Body weight was a weak determinant of serum 25OHD concentration in Korean men and women age 50 years and older. In contrast, higher body fat, for any given body weight, was a significant indicator of a lower serum 25OHD concentration in the men. These associations followed a similar pattern but were attenuated in the women. These findings indicate that body fat mass is a significant determinant of the serum 25OHD concentration, independent of body weight.

## Figures and Tables

**Table 1:** General characteristics of study subjects.

	Total (*n* = 6458)	Men (*n* = 3164)	Women (*n* = 3294)
Age (years)	61.5 (0.2)	60.8 (0.2)	62.3 (0.2)
Body weight (Kg)	62.1 (0.2)	66.8 (0.3)	56.9 (0.2)
Height (cm)	160.6 (0.1)	167.1 (0.1)	153.4 (0.1)
BMI (kg/m^2^)	24.0 (0.1)	23.9 (0.1)	24.2 (0.1)
Total body fat (Kg)	17.17 (0.11)	15.04 (0.15)	19.56 (0.13)
Trunk fat (Kg)	9.46 (0.07)	8.51 (0.09)	10.53 (0.08)
Limb fat (Kg)	6.77 (0.05)	5.53 (0.05)	8.17 (0.05)
Lean body mass (Kg)	44.44 (0.16)	51.12 (0.18)	36.86 (0.11)
25(OH)D (ng/mL)	20.2 (0.2)	21.6 (0.3)	18.6 (0.2)
Energy intake (kcal)	1883.7 (16.7)	2166.7 (21.7)	1566.4 (16.1)

Data are mean ± Standard error after data weighting. BMI; body mass index. Total body fat, Trunk fat, Limb fat are measured by Dual-Energy X-ray-Absorptiometry. 25(OH)D; 25-hyroxyvitamin D, Energy intake; daily total calorie intake.

**Table 2:** Partial correlation of serum 25(OH)D with body weight, height, body fat, and lean body mass.

	Serum 25(OH)D Men (*n* = 3164)	Serum (25(OH)D Women (*n* = 3294)
Body weight	−0.075 **	−0.073 **
Height	−0.052 *	−0.023
BMI	−0.058 *	−0.071 **
Waist	−0.043 *	−0.045 *
Total body fat	−0.154 **	−0.116 **
Trunk fat	−0.142 **	−0.108 **
Limb fat	−0.164 **	−0.111 **
Lean body mass	0.023	0.013

Values represent Partial Correlation Coefficient after age adjustment. * *p* < 0.05, ** *p* < 0.001. 25(OH)D, body weight, height, body fat, trunk fat, limb fat, and lean body mass measured by Dual-Energy X-ray-Absorptiometry.

**Table 3:** Comparison of variables by body weight tertile in both genders.

	Men Body Wt				Women Body Wt			
Variables	T1 (*n* = 1047)	T2 (*n* = 1064)	T3 (*n* = 1053)	*p* Value	T1 (*n* = 1099)	T2 (*n* = 1096)	T3 (*n* = 1099)	*p* Value
Age (years)	64.2 (0.4)	60.6 (0.3)	58.4 (0.3)	<0.001	65.6 (0.5)	61.0 (0.4)	60.4 (0.3)	<0.001
Body weight (Kg)	55.6 (0.2)	65.7 (0.1)	76.5 (0.2)	<0.001	47.8 (0.1)	56.3 (0.1)	66.0 (0.2)	<0.001
Height (cm)	163.3 (0.2)	166.9 (0.2)	170.3 (0.2)	<0.001	150.0 (0.2)	153.5 (0.2)	156.5 (0.2)	<0.001
BMI (kg/m^2^)	20.9 (0.1)	23.6 (0.1)	26.4 (0.1)	<0.001	21.3 (0.1)	24.0 (0.1)	27.0 (0.1)	<0.001
Total body fat (Kg)	10.70 (0.16)	14.74 (0.12)	18.72 (0.20)	<0.001	14.78 (0.15)	19.23 (0.12)	24.38 (0.15)	<0.001
Trunk fat (Kg)	5.73 (0.10)	8.32 (0.08)	10.89 (0.12)	<0.001	7.70 (0.10)	10.33 (0.08)	13.38 (0.10)	<0.001
Limb fat (Kg)	4.06 (0.06)	5.43 (0.05)	6.77 (0.08)	<0.001	6.27 (0.06)	8.03 (0.06)	10.08 (0.07)	<0.001
Lean body mass (Kg)	44.48 (0.17)	50.39 (0.13)	57.00 (0.20)	<0.001	32.74 (0.10)	36.59 (0.11)	40.99 (0.15)	<0.001
25(OH)D (ng/mL)	22.0 (0.4)	21.7 (0.3)	21.3 (0.3)	0.164	19.2 (0.3)	18.3 (0.3)	18.4 (0.3)	0.088
Energy intake (kcal)	2017.1 (30.0)	2134.5 (30.0)	2313.3 (39.3)	<0.001	1481.3 (22.6)	1589.1 (27.5)	1622.2 (25.8)	<0.001

Data are mean ± Standard Error after data weighting. *p* values are from general linear model in complex data analysis. T1, T2, T3 represent tertiles of body weight. BMI; body mass index. Total body fat, trunk fat, and limb fat are measured by Dual-Energy X-ray-Absorptiometry.
